# Hyponatremia upon presentation to the emergency department – the need for urgent neuroimaging studies

**DOI:** 10.1038/s41598-017-02030-6

**Published:** 2017-05-16

**Authors:** Arne Bokemeyer, Rainer Dziewas, Heinz Wiendl, Wolfram Schwindt, Paul Bicsán, Philipp Kümpers, Hermann Pavenstädt

**Affiliations:** 1 Department of Medicine D, Division of General Internal Medicine, Nephrology, and Rheumatology, University Hospital Münster, Albert-Schweitzer-Campus 1 A1, 48149 Münster, Germany; 2 Department of Neurology, University Hospital Münster, Albert-Schweitzer-Campus 1 A1, 48149 Münster, Germany; 3 Division of Information Technology - Clinical Systems, University Hospital Münster, Albert-Schweitzer-Campus 1 D5, 48149 Münster, Germany; 4 Department of Radiology, University Hospital Münster, Albert-Schweitzer-Campus 1 A1, 48149 Münster, Germany

## Abstract

This study aims to evaluate the necessity of urgent neuroimaging for emergency admissions exhibiting symptomatology of profound hyponatremia. We retrospectively analyzed the medical records of all patients admitted to the emergency room of the University Hospital Münster from 2010 to 2014 with a serum sodium value < 125 mmol/L. From 52918 emergency admissions, 261 patients with profound hyponatremia were identified, of whom 140 (54%) had neurological symptoms. Unspecific weakness and confusion were the most prevalent of these symptoms (59%). *Focal neurological signs* [*FNS*] were present in 31% of cases and neuroimaging was performed in 68% (95/140) of symptomatic patients. Multiple logistic regression analysis identified *FNS*, *seizures, altered consciousness* and *age* as independent predictors for conducting neuroimaging (all p < 0.05). Significant pathological findings consistent with acute symptomatology were evident in 17 cases, all of whom had *FNS. R*ecursive partitioning analyses confirmed *FNS* as the best predictor of neuroimaging pathology (p < 0.001). Absence of *FNS* had a negative predictive value of 100% [95% confidence interval: 93–100%] for excluding neuroimaging pathology. In conclusion, emergency patients with profound hyponatremia frequently show nonspecific-neurological symptoms and may undergo neuroimaging unnecessarily. The lack of *FNS* may serve as a valuable criterion for withholding neuroimaging until hyponatremia has been corrected.

## Introduction

Hyponatremia is one of the most common disorders of body fluid and electrolyte balance encountered in clinical practice, and is primarily a disorder of water balance, with a relative excess of body water compared to total body solutes^1–3^. Profound hyponatremia (sodium <125 mmol/L) affects approximately 1% of all emergency admissions and is associated with more than twice the risk of in-hospital mortality^1, 2, 4–6^. It may cause cellular swelling with brain edema and subsequent dysfunction of the central nervous system^1, 2, 7^.

The clinical symptoms of hyponatremia vary according to the speed of onset and the severity of electrolyte imbalance. Symptoms can range from mild to severe, beginning with *weakness/confusion* and progressing to *seizures* and *alteration of consciousness* (e.g. somnolence, stupor, coma)^2, 8^.

Current management practices concerning initial neurological diagnostics and the use of neuroimaging in patients with profound hyponatremia remain unclear^1^. Previous clinical reviews and recent interdisciplinary *European guidelines* suggest immediate treatment with hypertonic saline in patients with profound hyponatremia and severe symptoms^1, 2, 9^. If neurological symptoms do not improve after saline infusion, these guidelines vaguely state that “…additional neurological investigations such as imaging may be helpful…”^1^. We noticed that there is a low threshold for conducting urgent neuroimaging in symptomatic emergency patients, possibly due to a lack of specific recommendations and a multitude of neurological symptoms, although hyponatremia could be a good explanation for the symptoms of such patients’.

Therefore, the aim of the present study was to identify a pattern of neurological symptoms in which urgent neuroimaging is required and to determine whether there is any symptomatology in which neuroimaging could be safely postponed.

## Results

### Study population

**Figure 1:**
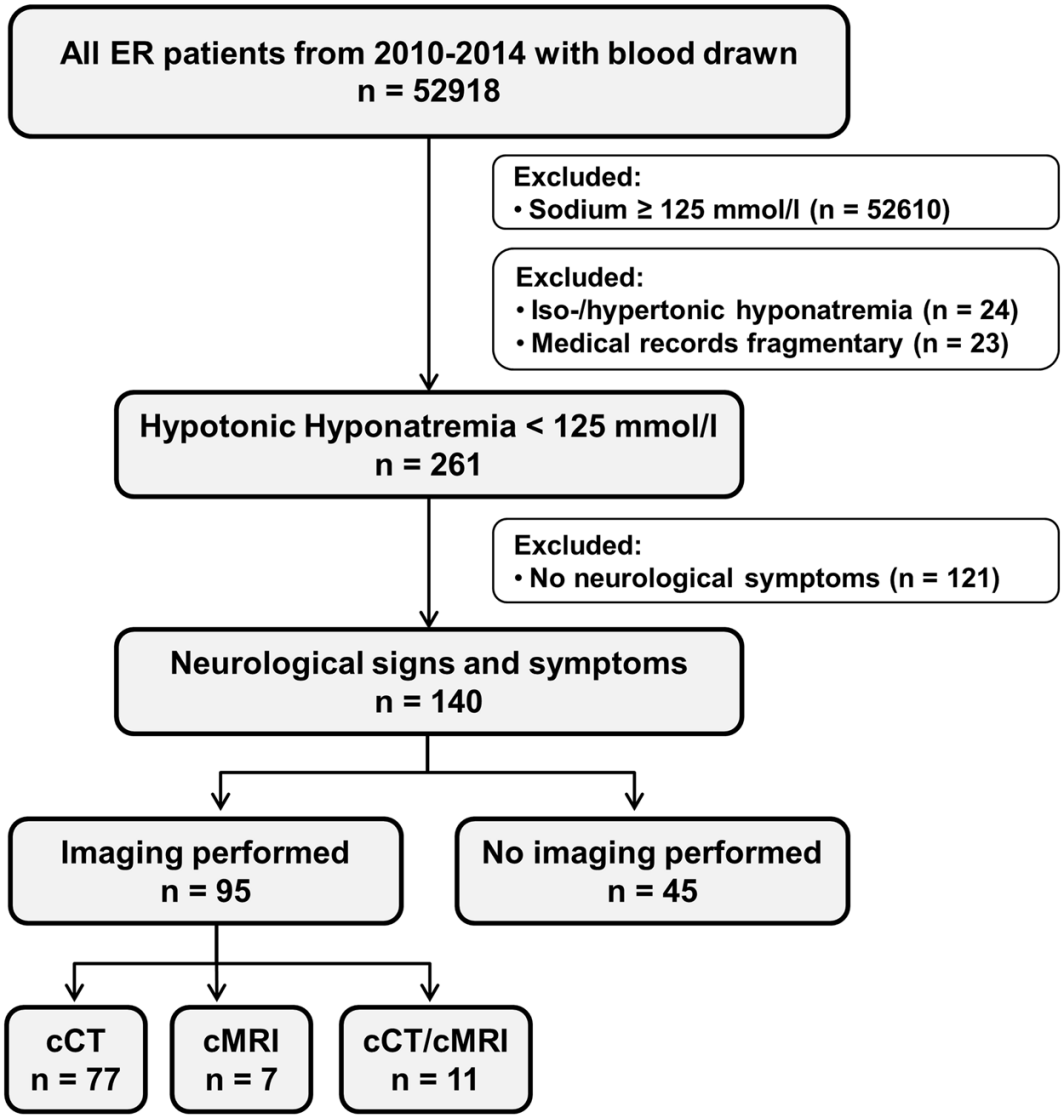
Participant flow chart of emergency admissions with profound symptomatic hyponatremia (University Hospital Münster, 2010–2014). ER, emergency room; cCT, cranial computed tomography; cMRI, cranial magnetic resonance tomography.

From 2010 to 2014, we identified 261 emergency department patients suffering from profound hypotonic hyponatremia (representing 0.49% of all emergency admissions during the study period). Following the exclusion of patients without neurological symptoms, the final dataset consisted of 140 patients with neurological symptoms consistent with symptomatic hyponatremia (53.6% of patients with profound hyponatremia) (Fig. 1). Patients were predominantly female (67.9%) and the median age was 66 years (range: 55–79 years). The mean serum sodium value was 120 mmol/L and mean plasma osmolality was 246 mmol/L (Table 1). Basic neurological assessment was documented in all patients by experienced emergency physicians. In addition, detailed neurological consultation reports were available for 75% of the patients with symptomatic hyponatremia.

**Table 1:** Characteristics of patients with severe hyponatremia and neurological symptoms.

Variables	All patients	Neuroimaging performed	No neuroimaging performed	P-Value
	(n = 140)	(n = 95)	(n = 45)	
Age, in years	66 (55–79)	73 (57–81)	61 (51–76)	**0.013**
Gender (female in %)	95 (67.9)	70 (73.7)	25 (55.5)	**0.032**
Vital Signs				
Systolic blood pressure, mmHg	130 (116–146)	140 (126–160)	120 (109–133)	**0.007**
Diastolic blood pressure, mmHg	80 (70–90)	80 (70–90)	75 (60–84)	0.115
Heart frequency/min	80 (67–90)	84 (72–91)	80 (66–90)	0.254
Laboratory Parameters, mmol/l				
Plasma sodium	120 (116–122)	121 (117–122)	119 (116–121)	0.134
Plasma osmolality	246 (236–257)	245 (236–256)	248 (237–261)	0.512
Urine sodium	47 (17–90)	62 (20–95)	37 (15–70)	**0.032**
Urine osmolality	294 (200–381)	321 (223–430)	252 (170–344)	**0.017**
Etiology of Hyponatremia, in %				
Diuretics	23.6	23.1	24.4	0.867
SIAD	31.4	29.4	33.3	0.956
Cortisol deficiency	5.7	4.2	8.9	0.120
Hypovolemia	20.0	17.9	26.7	0.232
Hypervolemia	6.4	2.1	15.5	**0.002**
Primary Polydipsia	7.9	8.4	6.7	0.880
Others	1.4	1.1	2.2	0.586
Neurological symptoms resolved upon hyponatremia treatment	124 (88.6)	79/95 (83.2)	45/45 (100.0)	**0.003**
pathological neuroimaging^*^		1/17 (5.9)		
non-pathological neuroimaging^#^		78/78 (100)		
Door-to-imaging-time, minutes		123 (61–238)		

Median and interquartile range reported for continuous variables and frequency; percentage reported for categorical variables. Differences between patients with/without neuroimaging were calculated using the Mann-Whitney U test for continuous variables and the Chi-square test for categorical variables. Two-sided *p* values < 0.05 were considered statistically significant. SIAD, syndrome of inappropriate antidiuresis (SIAD). *Pathological neuroimaging: neuroimaging showed findings related to acute symptomatology, e.g., ischemic stroke. ^#^Non-pathological neuroimaging: neuroimaging was unremarkable (no incidental/pathological finding) or showed incidental findings not related to acute symptomatology, e.g., general cerebral atrophy, old ischemic stroke.

### Neurological symptoms in hyponatremia

**Figure 2:**
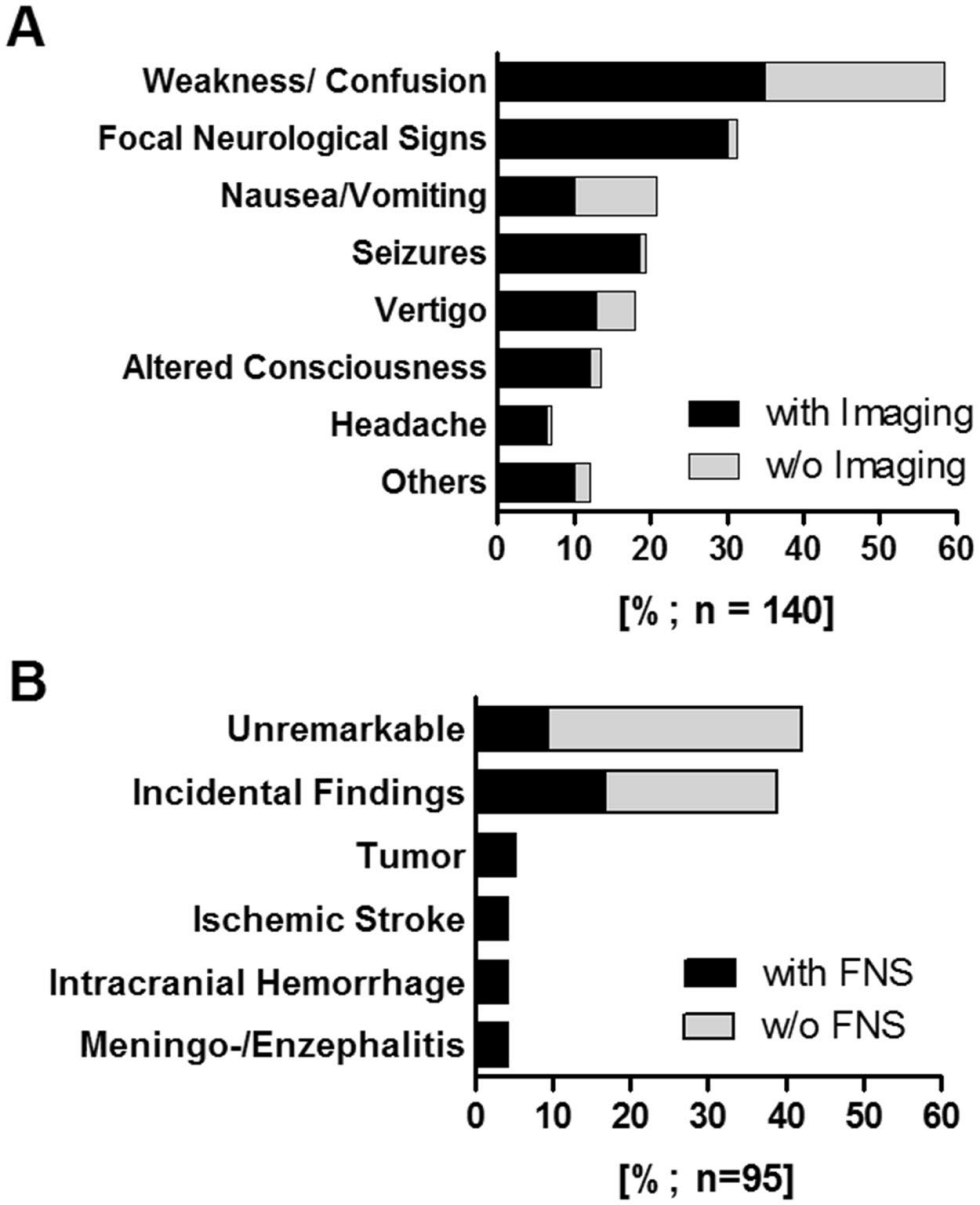
Neurological symptoms and imaging findings. (**A**) Prevalence of neurological symptoms (*focal neurological signs [FNS]* and *signs of global brain dysfunction*) in all patients with symptomatic hypotonic hyponatremia in our cohort (n = 140). (**B**) Results of neuroimaging studies (cCT and/or cMRI) in 95 of 140 patients (67.8%). Incidental findings (n = 38): imaging findings, which were not related to acute symptoms (multiple findings were permitted): general cerebral atrophy (n = 20), age-related periventricular white matter changes (n = 16), old ischemic stroke (n = 15), arteriosclerosis (n = 15), calcification of pineal gland/choroid plexus (n = 8), signs of former neurosurgical interventions (n = 3), signs of old fracture (n = 2).

Consistent with the literature, nonspecific weakness and confusion were the most prevalent symptoms (59%), followed by *focal neurological signs (FNS*, 31%) including cranial nerve palsy, hemiparesis, gaze palsy and hemiataxia (Fig. 2A).

### Predictors for conducting neuroimaging

Neuroimaging was performed frequently in patients with severe symptoms (*FNS*, *seizures* and *alteration of consciousness)*, whereas imaging was less frequently requested in other symptoms (Fig. 2A). Urgent neuroimaging studies (cCT and/or cMRI) were performed in 67.9% of patients (95/140). The majority of these (92.6%) were cCT scans performed at a median of 2 h after ER admission (Fig. 1). Using multivariate Cox regression analysis, we identified *age* and the presence of certain neurological symptoms (*seizures*, *alteration of consciousness* and *FNS)* as predictors for conducting neuroimaging studies (all p < 0.05) (Table 2).

**Table 2:** Predictors for conducting neuroimaging (cCT/cMRI) using logistic regression analysis.

	*Univariate*		*Multivariate*	
	OR (95% CI)	P-value	OR (95% CI)	P-value
Plasma sodium level	1.05 (0.98–1.13)	0.161		
Gender (female)	2.15 (1.02–4.51)	0.042	2.49 (0.90–6.93)	0.079
**Age**	1.31 (1.04–1.064)	0.018	1.64 (1.18–2.28)	**0.003**
Neurological symptoms				
Weakness/Confusion	0.37 (0.17–0.81)	0.013	0.59 (0.21–1.69)	0.325
Nausea/Vomiting	0.36 (0.15–0.83)	0.016	0.67 (0.22–2.05)	0.480
Headache	4.14 (0.50–34.12)	0.187		
Vertigo	1.30 (0.50–3.38)	0.587		
** Seizures**	17.0 (2.2–129.40)	0.006	71.7 (7.33–700.6)	**<0.001**
** Altered consciousness**	4.80 (1.06–21.72)	0.042	6.71 (1.14–39.46)	**0.035**
** Focal neurological signs**	17.43 (4.0–76.11)	0.001	32.95 (6.59–164.7)	**<0.001**

Odds ratios (OR), 95% confidence intervals (CI), and p-values were calculated using logistic regression analysis (backward elimination). Variables were selected a priori based upon theoretical considerations and existing literature. Factors statistically significant at the 10% level in our univariate analysis were then included in our multivariate model. Two-sided p values < 0.05 were considered statistically significant in the multivariate model.

### Imaging findings in hyponatremia and predictors for neuroimaging pathology

Neuroimaging was unremarkable, or showed incidental findings not related to acute symptomatology, in 82.1% of all patients. In line with this, the symptoms of all these patients resolved upon hyponatremia treatment (Table 1). However, in 17 out of 95 patients (17.9%), neuroimaging revealed brain tumors (5.3%), ischemic stroke (4.2%), intracranial hemorrhage (4.2%) and meningo-/encephalitis (4.2%) (Fig. 2B). Nearly all of these patients failed to respond to hyponatremia treatment (94.1%; Table 1).

All 17 of these patients showed acute *FNS* **(**Fig. 2B; Supplementary Table S2), which highlighted the role of *FNS* as a strong predictor for neuroimaging pathology. Additional logistic regression analyses did not reveal any other variable which could predict pathological neuroimaging (Supplementary Table S1). Furthermore, regression tree analysis (recursive partitioning) confirmed *FNS* as the best predictor for neuroimaging pathology (p < 0.001; Supplementary Fig. S1). The presence of *FNS* predicted relevant neuroimaging findings with a sensitivity of 100% (95% confidence interval (CI): 79–100%), and a specificity of 68% (95% CI: 63–68%) (Chi-square test: p < 0.0001). The positive predictive value was 41% (95% CI: 32–41%). Accordingly, the absence of *FNS* had a negative predictive value of 100% (95% CI: 93–100%) for excluding significant neuroimaging pathology.

The number needed to test (NNT) for conducting neuroimaging with the aim of revealing pathological findings in patients with symptomatic hyponatremia was 5.6 in our original retrospective cohort. In other words, one out of six patients scanned had a pathological neuroimaging finding. Keeping in mind that the absence of *FNS* might serve as a criterion for withholding neuroimaging, the NNT for conducting neuroimaging could have been improved to 2.6 if scanning had been limited to the patients with *FNS* only.

## Discussion

This retrospective study shows that in emergency admissions with profound hyponatremia, nonspecific-neurological symptoms are frequent and account for potentially unnecessary neuroimaging. A lack of specific recommendations in the current guidelines means that clinicians feel uncertain about the need for and timing of neuroimaging for symptomatic hyponatremia^1, 2, 10–12^. Since a high number (17.9%) of patients with profound hyponatremia in the present study showed neuroimaging abnormalities, we suggest careful consideration of neuroimaging for this group of patients (Fig. 2).

Importantly, we have identified *focal neurological signs (FNS)* as the best predictor for neuroimaging pathology with a negative predictive value of 100%. The absence of *FNS* might therefore serve as a criterion to rule out neuroimaging until hyponatremia has been corrected.

Our data reveal that the majority of patients with profound hyponatremia show non-specific symptoms such as weakness/confusion or nausea. However, only *FNS* was clearly associated with pathological neuroimaging findings, indicating that in patients with hyponatremia and *FNS* imaging is indispensable. This finding might be explained by differences in the pathophysiology of the signs and symptoms observed in such clinical situations. Hypotonic hyponatremia represents an excess of water relative to its solutes in the extracellular compartment. Because water can transfer freely from the extracellular to the intracellular compartment, hypotonic hyponatremia can lead to cellular swelling followed by brain edema. As this cellular swelling is not limited to one focal cerebral region, but affects the whole brain, it can result in generalized central nervous dysfunction^1, 2, 7^. In contrast to this, the presence of *FNS* indicates focal brain disturbances caused by various types of pathology (e.g. ischemic stroke)^13–16^. Accordingly virtually all of our patients with *FNS* and neuroimaging pathology did not respond to hyponatremia treatment.

Doctors, particularly in emergency settings require an easy-to-follow decision tree^11, 12^. Taking the absence of *FNS* as a simple rule for withholding neuroimaging in patients with profound and symptomatic hyponatremia would allow clinicians to focus on treating the hyponatremia^1^. However, patients with *FNS* should receive neuroimaging without delay, since in these patients an alternative etiology of symptoms other than hyponatremia is to be expected. If this rule had been applied in our cohort, only 44 imaging studies would have been performed instead of 95, and no relevant cerebral pathology would have been missed. Avoidance of unnecessary imaging would reduce radiation exposure, costs, and burden upon the emergency room and radiology resources. Potentially, we might have refused, or at least delayed, necessary neuroimaging in patients presenting without *FNS*, although this was not the case in our study. Of course, clinicians should consider the clinical context and may decide to proceed with direct imaging, or other diagnostic pathways as certain neurological conditions, including brain tumors, can have a prolonged course that may not lead to *FNS* evident on routine bedside examination. Furthermore, we recommend that neuroimaging should be carried out according to current guidelines in patients presenting with traumatic brain injury independent from hyponatremia.

Consistent with the literature, hyponatremia is frequent in patients suffering from any type of brain injury (e.g. traumatic brain injury or bleeding)^17, 18^. However, the pathophysiology of hyponatremia following brain injury is not completely understood, but is known to develop rapidly after brain injury and may often be caused by a sudden release of vasopressin [s*yndrome of inappropriate antidiuresis* (SIAD)] and to a lesser extent by *adrenocorticotropic hormone insufficiency* or *cerebral salt wasting syndrome* ^19–21^. Besides acute brain injury, chronic pain can also be a strong stimulus for vasopressin^1^. We could therefore speculate that our patients with neuroimaging pathology developed hyponatremia secondary to the cerebral process encountered.

Our study has several limitations. Firstly, this was a retrospective, single-center study and our final data set only involved 140 patients. However, we collected data over a five year period and we were able to collate a homogenous and complete data set featuring a significant number of detailed neurological reports. Secondly, caution should be exercised when extrapolating our findings from the emergency room to the in-patient ward, although we expect the pathophysiological concept of *FNS* versus generalized brain edema to be valid in almost any clinical scenario. Thirdly, 45 of the 140 patients with symptomatic hyponatremia did not undergo neuroimaging, so we cannot exclude preselection bias. However, extensive evaluation of electronic chart records and discharge letters revealed that acute symptomatology resolved in all of these patients upon treatment for hyponatremia, almost excluding a causative role of any other (undiscovered) neurological diseases in this group. Fourthly, most imaging studies were performed despite the knowledge of profound hyponatremia; however in seven out of 95 patients, neuroimaging was performed immediately after admission before laboratory results became available. Fifthly, although we intended to only include patients with acute hyponatremia (duration <48 h), we cannot exclude that we may have included single cases with chronic hyponatremia (duration >48 h). However, the acute onset of symptoms was ascertained in all patients. Furthermore, all patients responded immediately and were provided standard treatment for acute hyponatremia. Finally, a significant difference in urine sodium and urine osmolality distribution in patients receiving, and not receiving neuroimaging was revealed. This was possibly driven by the different distribution of etiology in hyponatremia (e.g., significantly more cases of hypervolemic hyponatremia in the non-neuroimaging group).

Collectively, our data indicate that neurological symptoms in emergency patients with profound hyponatremia are relatively common and can lead to unnecessary neuroimaging. With due care, a lack of *focal neurological signs* might serve as a criterion for safely withholding neuroimaging until hyponatremia has been corrected. To strengthen these recommendations from our single-center study, further research is needed in future.

## Methods

### Study design and patients

This retrospective study was performed in the multidisciplinary emergency department of the University Hospital Münster, in which approximately 15.000 (mostly internal medicine and neurological) emergency room patients are treated each year. This study was approved by the Ethics Board of the Westphalian Wilhelm-University of Münster and the Medical Council of Westphalia-Lippe [Germany] and was performed in accordance with the Helsinki declaration. As approved by the Ethics Board, informed patient consent was not required for this study because of its retrospective design. Data from all patients ≥18 years of age who had a routine blood test in the emergency department between 2010 and 2014 were retrieved from the hospital information system. Next, patients with a serum sodium level ≥125 mmol/L were excluded (n = 52610). As we were interested in hypotonic hyponatremia, and because this is also what guidelines tend to focus upon, we excluded all cases with hypertonic or isotonic hyponatremia (serum osmolality ≥280 mmol/L or serum glucose ≥200 mg/dL; n = 24)^1, 2^ and those with incomplete medical records (n = 23). Clinical and laboratory data as well as neuroimaging reports (cranial computed tomography [cCT] and magnetic resonance imaging [cMRI]) were extracted from electronic medical records. A flow chart showing the inclusion and exclusion criteria for our patients is given in Fig. 1.

### Neurological evaluation and definitions

Electronic chart records and discharge letters, including medical history, patient complaints, medication, detailed physical examination, laboratory parameters, diagnostics including imaging studies, and the time of hospital stay (cumulative period in the emergency department and hospital) were screened for neurological symptoms by the study team. Acute neurological symptoms preceding hospital admission (e.g., seizures, vomiting) and those presenting upon admission, were taken into account (multiple symptoms were permitted). We differentiated between *focal neurological signs* (*FNS*; e.g., cranial nerve palsy, hemiparesis, gaze palsy and hemiataxia) and *signs of global brain dysfunction* (e.g., alteration of consciousness, nausea/vomiting, weakness/confusion). Cases were thoroughly reviewed by at least two independent board certified investigators with longstanding clinical expertise in emergency medicine (AB, RD or PK) and neurological symptoms were only recorded, if a new onset of symptomatology could be ascertained, and/or symptoms were not already known for longer times to include only cases of acute hyponatremia. None of our patients had advanced underlying dementia, as ascertained by chart review.

### Neuroimaging

cCT and cMRI were undertaken upon request by the treating physician and performed according to standardized radiology department procedures. Imaging reports were retrospectively classified as follows: unremarkable (no incidental/pathological finding), incidental (incidental finding not related to acute symptoms, e.g., general cerebral atrophy or old ischemic stroke) or pathological (findings related to acute symptomatology, e.g., ischemic stroke).

### Statistical analysis

Data are presented as absolute values, percentages or medians with corresponding 25th and 75th percentiles (interquartile range; IQR) or 95% confidence intervals (CI), where stated. Differences between groups of patients were calculated using the Mann-Whitney U test for continuous variables and the Chi-square test for categorical variables. Predictors for conducting neuroimaging, and for the presence of neuroimaging pathology, were identified by logistic regression. Variables were selected *a priori* and were based upon theoretical considerations and existing literature^2^. Variables found to be statistically significant at the 10% level in our univariate analysis were then included in a multivariate model. Two-sided *p* values < 0.05 were considered statistically significant. In an exploratory approach, all variables incorporated in the multivariate cox logistic regression model were concurrently subjected to a decision tree analysis to recursively identify the best predictors and patient subgroups with different probabilities for neuroimaging. Data analysis was performed using IBM SPSS Statistics 22.0 (IBM Corp., Armonk, NY, USA). Contingency table-derived data and likelihood ratios were calculated using the StatPages website^22^. Figures were prepared using GraphPad Prism 5.02 (GraphPad Software, La Jolla, CA, USA).

## Electronic supplementary material

Supplementary Information
