# Supplementary material for: Hyponatremia upon presentation to the emergency department – the need for urgent neuroimaging studies
Source: Sci Rep. 2017 May 16;7:1953. doi: 10.1038/s41598-017-02030-6 (PMC5434027; doi:10.1038/s41598-017-02030-6)
Supplement: Supplementary file 1 — Supplementary Information [file 41598_2017_2030_MOESM1_ESM.pdf]

Hyponatremia upon presentation to the emergency department – the need for urgent neuroimaging studies

Arne Bokemeyer[1#] · Rainer Dziewas[2] · Heinz Wiendl[2] · Wolfram Schwindt[4] · Paul Bicsán[3] · Philipp Kümpers[1*] · Hermann Pavenstädt[1*]

*Authors contributed equally and are both considered senior authors

[1] Department of Medicine D, Division of General Internal Medicine, Nephrology, and Rheumatology, University Hospital Münster, Albert-Schweitzer-Campus 1 A1, 48149 Münster, Germany; [2] Department of Neurology, University Hospital Münster, Albert-Schweitzer-Campus 1 A1, 48149 Münster, Germany; [3] Division of Information Technology - Clinical Systems, University Hospital Münster, Albert-Schweitzer-Campus 1 D5, 48149 Münster, Germany; [4] Department of Radiology, University Hospital Münster, Albert-Schweitzer-Campus 1 A1, 48149 Münster, Germany. [#]Correspondence and requests for materials should be addressed to A.B. (email: arne.bokemeyer@ukmuenster.de)

Supplementary Table S1. Predictors for pathological neuroimaging findings by logistic regression analysis

	Univariate	
	**OR (95% CI)**	***P*-value**
Plasma sodium level	1.14 (0.97 – 1.35)	0.105
Gender (female)	0.83 (0.26 – 2.64)	0.749
Age	0.92 (0.66 - 1.28)	0.922
Neurological symptoms		
Weakness/Confusion	0.80 (0.28 – 2.30)	0.681
Nausea/Vomiting	1.31 (0.32 – 5.30)	0.709
Headache	0.00	0.999
Vertigo	0.52 (0.11 – 2.49)	0.411
Seizures	0.00	0.999
Reduced state of consciousness	0.98 (0.25 – 3.87)	0.977
Focal neurological signs*		

Odds ratios (OR), 95% confidence intervals (CI), and p-values were calculated by logistic regression analysis (backward elimination). Variables were selected *a priori* based on theoretical considerations and existing literature. Those statistically significant at the 10% level in the univariate analysis were included in the multivariate model; two-sided *p* values <0.05 were considered statistically significant in the multivariate model.

* All patients with neuroimaging pathology had *focal neurological signs* (*FNS*); it was therefore statistically impossible to calculate these OR and p-values for *FNS* by logistic regression analysis.

Supplementary Table S2: All cases of patients with neuroimaging pathology related to acute symptomatology.

**No. of pat.**	**Sex**	**Age**	**Serum-Na** in mmol/l	**Symptomatology**	**Imaging findings**	**Diagnosis**	**Etiology of hyponatremia** (for all association with cerebral process possible [e.g. SIAD])
1	M	55	124	FNS – unilateral arm paralysis	Ischemic media infarction	Ischemic media stroke	Diuretics
2	F	32	123	FNS – brachiofaziale hemiparalysis	Encephalitis, multiple embolic infarctions	Endocarditis with septic encephalitis	Hypovolemia
3	F	78	120	FNS – unilateral gaze paralysis, pos. pyramidal sign; reduced state of vigilance	Encephalitis	Herpesencephalitis	Diuretics
4	F	41	123	FNS – leg paralysis	Tumor	Malig. melanoma with cerebral metastases	/
5	F	78	120	FNS – aphasia; nausea/vomiting; vertigo/falls	Ischemic media infarction	Ischemic media stroke	Hypervolemia
6	F	85	115	FNS – gait ataxia; weakness/confusion; nausea/vomiting; vertigo/falls	Intracranial hemorrhage	Intracranial hemorrhage caused by head injury	Diuretics
7	F	82	121	FNS – hemiparalysis, aphasia	Ischemic media infarction	Ischemic media stroke	/
8	M	61	123	FNS – unilateral gaze evoked nystagmus; nausea/vomiting	Tumor	Bronchial-CA with cerebral metastases	/
9	M	86	121	FNS – hemiparalysis, dysarthria	Tumor	Mamma-CA with cerebal metastases	/
10	F	90	124	FNS – aphasia	Tumor	Primary cerebral tumor	Neuroleptic drug
11	M	79	120	FNS – hemiparalysis; weakness/confusion	Intracranial hemorrhage and epidural bleeding	Intracranial hemorrhage and epidural bleeding	Neuroleptic drug
12	M	58	118	FNS – diploic images; weakness/confusion	Tumor of maxillary sinus with orbital infiltration	CA of maxillary sinus with orbital infiltration	/
13	F	43	115	FNS – anisocoria; reduced state of vigilance	Intracranial hemorrhage	Intracranial hemorrhage	/
14	F	79	123	FNS – hemihypoaesthesia, aphasia	Ischemic media infarction	Ischemic media stroke	/
15	F	66	124	FNS – aphasia, weakened tendon reflexes of legs; reduced state of vigilance	Subdural hematoma	Subdural hematoma	Diuretics, hypovolemia
16	F	65	117	FNS – clearly reduced unilateral arm reflex; neck pain; weakness/confusion	Cervical spondylodiscitis	Cervical spondylodiscitis	Hypovolemia
17	F	56	123	FNS – unilateral hearing loss; weakness/confusion	Meningitis, mastoiditis; tumor in sella turcica	Meningitis following mastoiditis	Addison disease

Generally in patients with cerebral processes, hyponatremia might be caused by SIAD (*syndrome of inappropriate antidiuresis*), *adrenocorticotropic hormone* (ACTH) *deficiency* and *cerebral salt wasting*. Therefore only additional possible causes for hyponatremia are enlisted in “Etiology of hyponatremia

Supplementary Figure S1: Prediction model by recursive partitioning analysis.

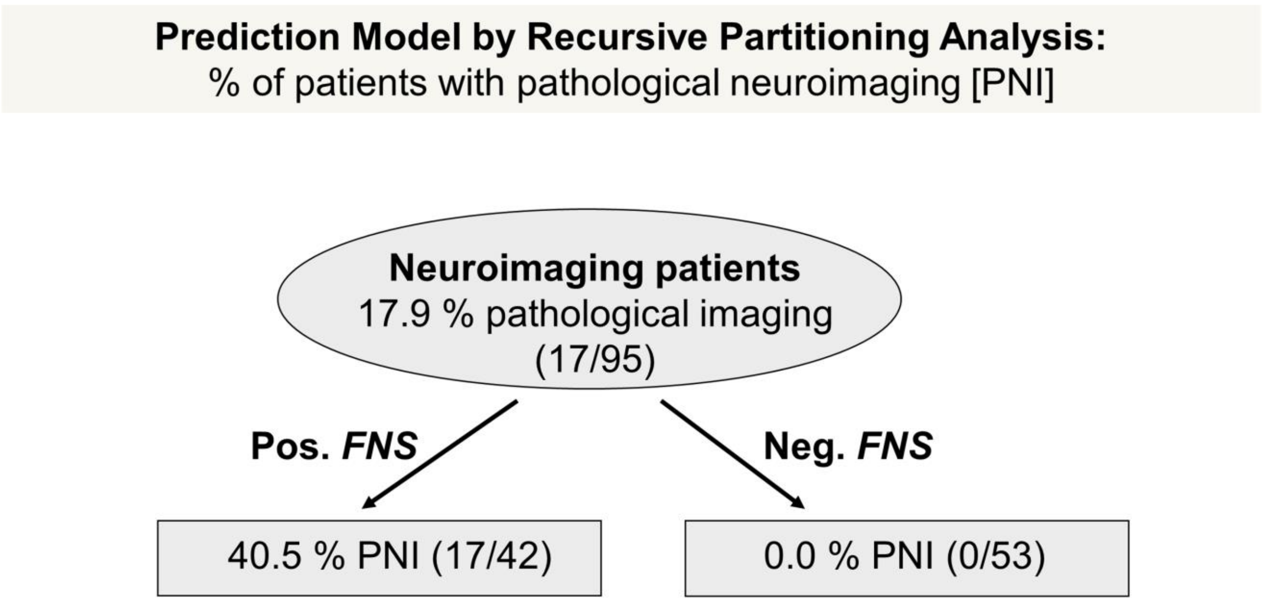

Two split variables. Percent of patients with neuroimaging pathology. *FNS–focal neurological signs.*
